# Species Delimitation and Phylogenetic Relationships in Ectobiid Cockroaches (Dictyoptera, Blattodea) from China

**DOI:** 10.1371/journal.pone.0169006

**Published:** 2017-01-03

**Authors:** Yanli Che, Shunhua Gui, Nathan Lo, Andrew Ritchie, Zongqing Wang

**Affiliations:** 1 College of Plant Protection, Southwest University, Beibei, Chongqing, P. R. China; 2 School of Life and Environmental Sciences, University of Sydney, Sydney, NSW, Australia; Sichuan University, CHINA

## Abstract

We collected Ectobiidae cockroach specimens from 44 locations in the south of the Yangtze valley. We obtained 297 COI sequences specimens and carried out phylogenetic and divergence dating analyses, as well as species delimitation analysis using a General Mixed Yule Coalescent (GMYC) framework. The intraspecific and interspecific sequence divergence in Ectobiidae cockroaches ranged from 0.0 to 7.0% and 4.6 to 30.8%, respectively. GMYC analysis resulted in 53 (confidence interval: 37–65) entities (likelihood ratio = 103.63) including 14 downloaded species. The COI GMYC groups partly corresponded to the ectobiid species and 52 ectobiid species were delimited successfully based on the combination of GMYC result with morphological information. We used the molecular data and 6 cockroach fossil calibrations to obtain a preliminary estimate of the timescale of ectobiid evolution. The major subfamilies in the group were found to have diverged between ~125–110 Ma, and morphospecies pairs were found to have diverged ~10 or more Ma.

## Introduction

Cockroach species are often difficult to differentiate, both at the adult and juvenile stages. Individuals of closely related species are often very morphologically similar [1–3]. Cockroaches display high developmental stochasticity, which results in great variation in external spination, setation and coloration [4,5], making it difficult to distinguish species on the basis of morphological characters. The male genitalia is of great value in the discrimination of male adult cockroaches; but for some closely related species, it is also very challenging (Zheng *et al*. [6], Che, Y.L., personal observation). Most taxonomic keys for cockroaches are based on adult male genitalia, which means that the females of closely related cockroaches cannot easily be matched with males of the same species, or females may appear to be entirely different species (Wang, Z.Q., personal observation). More importantly, juveniles may comprise up to 80% (Wang, Z.Q., personal observation) or 90% of individuals in most cockroach surveys [7]. Individuals may be highly polymorphic over the course of development and adults are often significantly different from juveniles [8,9]. The difficulty in distinguishing different developmental stages within a species and the nymphs of different species from each other makes it difficult to identify young developmental stages during field studies [4]. Consequently, simple, accurate and easily applicable methods are needed to facilitate the identification of cockroaches.

Since Hebert *et al*. [10,11] proposed the concept of DNA barcoding- a short standardized 658 bp fragment of the 5’end of the mitochondrial cytochrome oxidase-I [12] gene, it has proven to be a powerful tool to aid the identification of many insect species, including Lepidoptera [13–15], Coleoptera [16,17], Hemiptera [18], Hymenoptera [19,20], and Diptera [21–23]. Dai *et al*. [24] found the standard COI barcode outperformed two nuclear ITS genes in five different analytical methods that they implemented. And there has been some debate over its utility for species identification in some animal groups (Orthoptera: [25]; Lepidoptera: [26]). However, multiple independent datasets (i.e., DNA sequences and morphological evidence) to delimit species could help to solve it [27,28].

The generalized mixed Yule-coalescent (GMYC) model [29] has become one of the most popular approaches for species delimitation based on single-locus data. It models inter-species branching event as a Yule process [30,31], and intra-species branching events as a neutral coalescent process [32]. It then identifies the transition points between inter- and intra-species branching rates on a time-calibrated ultrametric tree by maximizing the likelihood score of the model. The Generalized Mixed Yule Coalescent (GMYC) model has been widely used in DNA barcoding studies, and has been shown to work well to delimit species in different groups [24,33–36].

To date, the application of molecular methods to aid in the identification and delimitation of cockroach species has been fairly limited. Knebelsberger & Miller [37] used COI sequences to distinguish three conspecific morphotypes of *Phyllodromica iberica* and infer phylogenetic relationships between the species of the *subaptera*-group. Evangelista *et al*. [38] used COI sequences to confirm the presence of a new invasive cockroach pest, *Periplaneta japonica* Karny, in New York. Yue *et al*. [39] confirmed that macropterous and brachypterous individuals of both sexes of *Hebardina concinna* were the same species using DNA barcodes. Evangelista *et al*. [5] used both morphological and genetic barcode information to estimate Blattodea species richness, and emphasized the importance of using independent datasets to delimit species boundaries and expert identification of specimens when possible. Similarly, in order to delimit species accurately, Ritchie *et al*. [40] suggested the combination of GMYC-based methods with other lines of evidence (morphology, ecology and developmental traits). There have been few studies that have examined a wide selection of cockroach taxa to determine whether results from molecular sequence based delimitation tools such as GMYC are consistent with designations based on traditional morphology. In this study, a combination of newly generated and publically available molecular data has been used to investigate the utility of GMYC methods and morphological evidence in understanding species limits among ectobiid cockroaches.

The cockroach family Ectobiidae is the most diverse cockroach family, containing some 2300 species [41]. The majority of ectobiid species are found in dead leaves or rotting logs of tropical and temperate forests, where they act as important natural decomposers. About 10 ectobiid species are important domiciliary pests and frequently found in human dwellings [42]. The most well known of these is *Blattella germanica*, which may pose certain health risks, including acting as a vector for pathogen transmission [43], and causing asthma [44,45]. We also performed molecular clock analyses to provide the first examination of the timeframe of evolution for ectobiid cockroaches.

## Material and Methods

### Specimen collection

**Fig 1:**
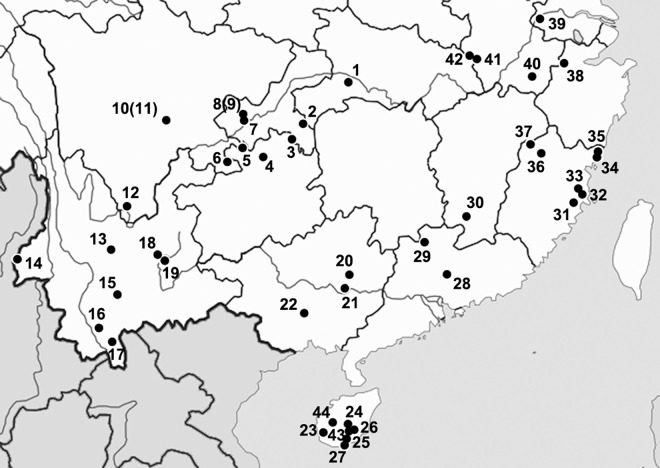
Distribution and collection localities of analyzed specimens of Ectobiidae in China. Numbers for sampling localities are as indicated in Table 1. Reprinted from [57] under a CC BY license, with permission from [Chao LI] original copyright [2016].

We collected more than one thousand Ectobiidae cockroach specimens from 44 sampling locations (Table 1, Fig 1) mainly in the south of China. Cockroaches were collected mainly by net, as well as by light trapping and canopy fogging (by Guo ZHENG, Institute of Zoology, Chinese Academy of Sciences). The collected insects were stored in 95% or 100% ethanol.

**Table 1:** 

Species	No. of location	location	Accession Number (Specimen voucher)
*Allacta ornata*	25	Diaoluo Mountain, Lingshui, Hainan (18°43', 109°52')	KY349665(AllaOrna1)
*Anaplectoidea spinea*	1	Nabang, Yinjiang, Yunnan (24°45', 97°34')	KY349591(AnapoSpi1), KY349589(AnapoSpi2), KY349590(AnapoSpi3)
*Anaplectoidea varia*	9	Emei Mountain, Leshan, Sichuan (29°34', 103°26')	KY349575(AnapoVar1), KY349573(AnapoVar2), KY349577(A1710_13(f))
	36	Tongmu, Wuyishan, Fujian (27°45', 117°41')	KY349579(AnapoVar3), KY349580(A1710_32(f))
	11	Jinyun Mountain, Beibei, Chongqing (29°50', 106°24')	KY349576(AnapoVar7), KY349578(A1710_72), KY349574(A1710_73)
	19	Shengtang Mountain, Jinxiu, Guangxi (23°58', 110°07')	KY349581(AnapoVar5), KY349582(A1710_52(f)), KY349583(A1710_53)
	22	Jianfengling Forest Park, Ledong, Hainan (18°44', 108°50')	KY349572(AnapoVar4), KY349571(AnapoVar6)
*Balta jinlinorum*	35	Jukou, Jianyang, Fujian (27°22', 117°57')	KY349669(BaltJinl2), KY349668(A33_22)
	20	Longtan Forest Park, Guiping, Guangxi (23°31', 109°59')	KY349666(BaltJinl3)
	36	Tongmu, Wuyishan, Fujian (27°45', 117°41')	KY349667(A2810_12(f))
*Blattella bisignata*	8	Dahei Mountain, Panzhihua, Sichuan (26°40', 101°43')	KY349776(BlatBisi1), KY349777(BlatBisi3), KY349778(A109_13(f))
	23	Wuzhishan Nature Reserve, Wuzhishan, Hainan (18°53', 109°40')	KY349774(BlatBisi4), KY349775(A109_42)
	4	Mojiang, Pu'er, Yunnan (23°21', 101°33')	KY349779(BlatBisi5), KY349780(A109_52(f)), KY349781(A109_53(f))
	32	Qishan Forest Park, Fuzhou, Fujian (26°02', 119°19')	KY349791(BlatBisi6), KY349792(BlatBisi7)
	21	Nanhu Park, Nanning, Guangxi (22°48', 108°21')	KY349789(BlatBisi8), KY349785(A109_82(f)), KY349786(A109_83(f))
	20	Longtan Forest Park, Guiping, Guangxi (23°31', 109°59')	KY349784(BlatBisi9), KY349787(A109_92(f)), KY349788(A109_93(f))
	3	Dadugang, Xishuangbanna, Yunnan (22°20', 100°55')	KY349790(BlatBisi10), KY349782(A109_102)
	2	Menglun, Xishuangbanna, Yunnan (21°56',1 01°15')	KY349783(A4109_11(f))
*Blattella germanica*	13	Chongqing University, Shapingba, Chongqing (29°34', 106°28')	KY349771(BlatGerm2), KY349772(BlatGerm3)
	9	Emei Mountain, Leshan, Sichuan (29°34', 103°26')	KY349773(BlatGerm4(f))
	4	Mojiang, Pu'er, Yunnan (23°21', 101°33')	KY349767(BlatGerm5(f))
	1	Nabang, Yinjiang, Yunnan (24°45', 97°34')	KY349768(BlatGerm6), KY349769(A1109_62), KY349770(A1109_63)
	3	Dadugang, Xishuangbanna, Yunnan (22°20', 100°55')	KY349766(A109_103)
*Blattella lituricollis*	33	Sansha, Xiapu, Fujian (26°55', 120°13')	KY349757(BlatLitu1), KY349760(BlatLitu2), KY349758(BlatLitu3), KY349756(A6009_13)
	25	Diaoluo Mountain, Lingshui, Hainan (18°43', 109°52')	KY349764(BlatLitu4), KY349756(A6009_43)
	29	Nanshan Village, Nankang, Jiangxi (25°38', 114°45')	KY349761(BlatLitu5), KY349762(A6009_52), KY349763(A6009_53)
	23	Wuzhishan Nature Reserve, Wuzhishan, Hainan (18°53', 109°40')	KY349765(A109_43(f))
*Blattella nipponica*	14	Simian Mountain, Jiangjin, Chongqing (28°39', 106°24')	KY349810(BlatNipp1(f))
	33	Sansha, Xiapu, Fujian (26°55', 120°13')	KY349807(BlatNipp2), KY349808(A6009_12),
	17	Zhuoshui,Qiangjiang, Chongqing (29°18',108°46')	KY349798(BlatNipp3), KY349801(BlatNipp4), KY349799(BlatNipp5), KY349800(BlatNipp11(f)), KY349803(BlatNipp13(f))
	27	Nankunshan Forest Park, Huizhou, Guangdong (23°37', 113°51')	KY349812(BlatNipp6)
	39	Fuxi Village, Huangshan, Anhui (30°04', 118°09')	KY349806(BlatNipp7)
	31	Shizhu Mountain, Fuqing, Fujian (25°43', 119°18')	KY349796(BlatNipp8(n))
	38	Zijin Mountain, Nanjing, Jiangsu (32°04', 118°51')	KY349793(BlatNipp9), KY349794(A5909_92), KY349795(A5909_93)
	16	Kuankuoshui Nature Reserve, Guiyang, Guizhou (28°14',107°12')	KY349811(BlatNipp10(f)), KY349809(A5909_103)
	40	Taohuachong Forest Park, Yingshan, Hubei (30°59', 116°01')	KY349802(BlatNipp12), KY349804(A5909_122), KY349805(A5909_123)
	32	Qishan Forest Park, Fuzhou, Fujian (26°02', 119°19')	KY349797(A109_63)
*Blattella radicifera*	2	Menglun, Xishuangbanna, Yunnan (21°56',1 01°15')	KY349677(BlatRadi1), KY349676(122_2), KY349678(122_1)
*Blattella sauteri*	37	Tianmushan Natural Reserve, Lin'an, Zhejiang (30°20', 119°25')	KY349682(BlatSaut1), KY349680(BlatSaut3), KY349683(BlatSaut7(f))
	30	Jiulonggu Forest Park, Putian, Fujian (25°26', 118°50')	KY349679(BlatSaut2)
	31	Shizhu Mountain, Fuqing, Fujian (25°43', 119°18')	KY349681(BlatSaut4), KY349684(A3409_42)
*Blattella* sp.1	39	Fuxi Village, Huangshan, Anhui (30°04', 118°09')	KY349686(BlatSp11), KY349687(62A5_1), KY349685(A3409_63)
	39	Fuxi Village, Huangshan, Anhui (30°04', 118°09')	KY349688(A5909_72(f))
*Blattella singularis*	26	Hongshulin Park, Sanya, Hainan (18°15', 109°30')	KY349753(BlatSing1), KY349754(BlatSing2), KY349755(BlatSing3(f)), KY349751(BlatSing4(n))
	3	Dadugang, Xishuangbanna, Yunnan (22°20', 100°55')	KY349752(BlatSing5(f))
*Episymploce conspicua*	37	Tianmushan Natural Reserve, Lin'an, Zhejiang (30°20', 119°25')	KY349741(EpisCons1), KY349746(EpisCons3), KY349742(EpisCons4)
	36	Tongmu, Wuyishan, Fujian (27°45', 117°41')	KY349743(EpisCons5), KY349744(A1009_52(f)), KY349745(A1009_53)
*Episymploce hunanensis*	28	Nanling Forest Park, Shaoguan, Guangdong (24°55', 113°05')	KY349727(EpisHuna2)
	17	Zhuoshui, Qiangjiang, Chongqing (29°18', 108°46')	KY349728(EpisHuna4), KY349729(EpisHuna5)
*Episymploce kunmingi*	5	Jindian Forest Park, Kunming, Yunnan (25°05', 102°46')	KY349718(EpisKunm1), KY349719(EpisKunm2), KY349720(EpisKunm3)
	6	Xiaotuan Mountain, Kunming, Yunnan (24°45', 103°25')	KY349721(EpisKunm4), KY349722(EpisKunm5), KY349723(EpisKunm6)
*Episymploce mamillatus*	7	Zixi Mountain, Chuxiong, Yunnan (24°52', 101°19')	KY349724(EpisMami1), KY349725(EpisMami2), KY349726(EpisMami3)
*Episymploce potanini*	42	Hejiaping, Changyang, Hubei (30°17', 110°34')	KY349747(EpisPota1), KY349749(EpisPota2), KY349748(EpisPota3), KY349750(EpisPota4(f))
	34	Taimu Mountain, Fuding, Fujian (27°11', 119°57')	KY349734(EpisPota6(f)), KY349737(A0510_62), KY349735(A0510_63)
	37	Tianmushan Natural Reserve, Lin'an, Zhejiang (30°20', 119°25')	KY349736(EpisPota5)
*Episymploce sinensis*	12	Southwest University, Beibei, Chongqing (29°49', 106°26')	KY349708(EpisSine1), KY349709(EpisSine2), KY349710(EpisSine3(f))
	42	Hejiaping, Changyang, Hubei (30°17', 110°34')	KY349712(EpisSine4)
	38	Zijin Mountain, Nanjing, Jiangsu (32°04', 118°51')	KY349716(EpisSine5)
	34	Taimu Mountain, Fuding, Fujian (27°11', 119°57')	KY349714(EpisSine6(f)), KY349713(EpisSine7(f)), KY349715(A0310_63(f))
	14	Simian Mountain, Jiangjin, Chongqing (28°39', 106°24')	KY349711(EpisSine8(f))
*Episymploce kryzhanovshii*	37	Tianmushan Natural Reserve, Lin'an, Zhejiang (30°20', 119°25')	KY349740(EpisSp11), KY349738(EpisSp12), KY349739(A0510_52)
*Episymploce* sp.2	8	Dahei Mountain, Panzhihua, Sichuan (26°40', 101°43')	KY349730(EpisSp21), KY349731(EpisSp22)
*Episymploce* sp.4	10	Shengli Village, Leshan, Sichuan (29°17', 103°01')	KY349717(EpisSp41)
*Episymploce spinosa*	8	Dahei Mountain, Panzhihua, Sichuan (26°40', 101°43')	KY349732(EpisSpin2), KY349733(EpisSpin3)
*Hemithyrsocera marginalis*	2	Menglun, Xishuangbanna, Yunnan (21°56', 101°15')	KY349662(HemiMarg1), KY349663(HemiMarg2)
	3	Dadugang, Xishuangbanna, Yunnan (22°20', 100°55')	KY349664(HemiMarg3(f))
*Hemithyrsocera vittata*	28	Nanling Forest Park, Shaoguan, Guangdong (24°55', 113°05')	KY349565(HemiVitt1), KY349566(HemiVitt3)
	20	Longtan Forest Park, Guiping, Guangxi (23°31', 109°59')	KY349567(HemiVitt4)
*Malacccina sinica*	24	Qixianling Forest Park, Baoting, Hainan (18°41', 109°40')	KY349584(MalaSini1(f)), KY349585(MalaSini2(n)), KY349586(MalaSini3(f)), KY349587(MalaSini4), KY349588(MalaSini5)
*Margattea bisignata*	28	Nanling Forest Park, Shaoguan, Guangdong (24°55', 113°05')	KY349600(MargBisi1), KY349601(MargBisi2), KY349602(MargBisi3)
	39	Fuxi Village, Huangshan, Anhui (30°04', 118°09')	KY349604(MargBisi11)
	20	Longtan Forest Park, Guiping, Guangxi (23°31', 109°59')	KY349605(MargBisi6)
	19	Shengtang Mountain, Jinxiu, Guangxi (23°58', 110°07')	KY349606(MargBisi7)
	41	Qingtaiguan, LuoTian, Hubei (31°11', 115°41')	KY349597(MargBisi8), KY349598(A1910_82)
	11	Jinyun Mountain, Beibei, Chongqing (29°50', 106°24')	KY349603(MargBisi9), KY349601(A1910_92)
	9	Emei Mountain, Leshan, Sichuan (29°34', 103°26')	KY349594(MargBisi4), KY349595(MargBisi5), KY349596(MargBisi10)
	35	Jukou, Jianyang, Fujian (27°22', 117°57')	KY349607(A33_33)
*Margattea concava*	22	Jianfengling Forest Park, Ledong, Hainan (18°44', 108°50')	KY349647(MargConc1), KY349648(MargConc3), KY349649(MargConc6(n))
	23	Wuzhishan Nature Reserve, Wuzhishan, Hainan (18°53', 109°40')	KY349650(MargConc4), KY349651(A2710_42)
	25	Diaoluo Mountain, Lingshui, Hainan (18°43', 109°52')	KY349652(MargConc5)
*Margattea multipunctata*	2	Menglun, Xishuangbanna, Yunnan (21°56', 101°15')	KY349645(MargMult1), KY349646(A4210_13(f))
*Margattea angusta*	152419	Sunzi Mountain, Gulin, Sichuan (28°11', 105°47')（应为吊罗山Qixianling Forest Park, Baoting, Hainan (18°41', 109°40')Shengtang Mountain, Jinxiu, Guangxi (23°58', 110°07')	KY349621(MargAngu1)KY349622(MargAngu2), KY349623(MargAngu3)KY349624(MargAngu4)
*Margattea nimbata*	12	Southwest University, Beibei, Chongqing (29°49', 106°26')	KY349654(MargNimb1), KY349658(MargNimb2), KY349655(MargNimb3), KY349653(MargNimb4), KY349657(MargNimb6(f))
	36	Tongmu, Wuyishan, Fujian (27°45', 117°41')	KY349656(MargNimb5(f))
*Margattea* sp.1	4	Mojiang, Pu'er, Yunnan (23°21', 101°33')	KY349659(MargSp11(f)), KY349661(A2210_12), KY349660(A2210_13(f))
*Margattea* sp.3	15	Sunzi Mountain, Gulin, Sichuan (28°11', 105°47')	KY349608(MargSp31), KY349609(MargSp32)
*Margattea speciosa*	37	Tianmushan Natural Reserve, Lin'an, Zhejiang (30°20', 119°25')	KY349620(MargSpec2)
	22	Jianfengling Forest Park, Ledong, Hainan (18°44', 108°50')	KY349618(MargSpec3)
	19	Shengtang Mountain, Jinxiu, Guangxi (23°58', 110°07')	KY349619(A2810_62)
*Margattea spinifera*	35	Jukou, Jianyang, Fujian (27°22', 117°57')	, KY349644(AF2010_2), KY349642(AF2010_3)
	30	Jiulonggu Forest Park, Putian, Fujian (25°26', 118°50')	KY349638(MargSpin2), KY349639(MargSpin3), KY349636(A2810_23)
	20	Longtan Forest Park, Guiping, Guangxi (23°31', 109°59')	KY349627(MargSpin7), KY349628(MargSpin8), KY349629(A2809_52), KY349630(62A1_1)
	32	Qishan Forest Park, Fuzhou, Fujian (26°02', 119°19')	KY349632(MargSpin9), KY349641(A2810_92), KY349633(A2810_93)
	36	Tongmu, Wuyishan, Fujian (27°45', 117°41')	KY349631(MargSpin10), KY349637(MargSpin1), KY349643(A2810_13)
	25	Diaoluo Mountain, Lingshui, Hainan (18°43', 109°52')	KY349640(A2710_53)
	34	Taimu Mountain, Fuding, Fujian (27°11', 119°57')	KY349634(MargSpin11), KY349635(A2810_112(f))
*Margattea* sp.5	20	Longtan Forest Park, Guiping, Guangxi (23°31', 109°59')	KY349625(MargSp51), KY349626(62A1_2)
*Margattea spinosa*	24	Qixianling Forest Park, Baoting, Hainan (18°41', 109°40')	KY349610(MargSpiA3), KY349611(MargSpiA5), KY349612(MargSpiA6),KY349617(MargSpiA4(f))
	21	Nanhu Park, Nanning, Guangxi (22°48', 108°21')	KY349613(MargSpiA8), KY349615(A2810_52), KY349614(A2810_53)
	25	Diaoluo Mountain, Lingshui, Hainan (18°43', 109°52')	KY349616(MargSpiA7)
*Shelfordina volubilis*	25	Diaoluo Mountain, Lingshui, Hainan (18°43', 109°52')	KY349562(ShelVolu1), KY349563(ShelVolu2), KY349564(ShelVolu3)
*Sigmella puchihlungi*	25	Diaoluo Mountain, Lingshui, Hainan (18°43', 109°52')	KY349523(SigmPuch2), KY349525(61_2)
	43	Liupancun, Jiyangzhen, Sanya, Hainan (N 18°14.8′ E 109°37.5′)	KY349529(61_4), KY349528(A6110_42), KY349530(A6110_43)
	44	Bawangling, Hainan (N 19°05.2′ E 109°07.3′)	KY349526(61_5), KY349527(61_6), KY349524(A6110_53)
*Sigmella schenklingi*	34	Taimu Mountain, Fuding, Fujian (27°11', 119°57')	KY349555(SigmSche5(f)), KY349554(A209_52(f)), KY349561(A209_53(f))
	18	Gongtan, Youyang, Chongqing (28°56', 108°21')	KY349558(SigmSche7(f)), KY349553(A209_62), KY349553(A209_63)
	39	Fuxi Village, Huangshan, Anhui (30°04', 118°09')	KY349556(SigmSche11), KY349557(SigmSche14)
	9	Emei Mountain, Leshan, Sichuan (29°34', 103°26')	KY349541(SigmSche4), KY349543(A209_42), KY349559(A209_43)
	40	Taohuachong Forest Park, Yingshan, Hubei (30°59', 116°01')	KY349545(SigmSche9), KY349540(SigmSche18), KY349539(A209_93)
	39	Fuxi Village, Huangshan, Anhui (30°04', 118°09')	KY349548(SigmSche16
	18	Gongtan, Youyang, Chongqing (28°56', 108°21')	KY349544(SigmSche6)
	17	Zhuoshui,Qiangjiang, Chongqing (29°18', 108°46')	KY349546(SigmSche12), KY349547(SigmSche13), KY349542(A209_14)
	14	Simian Mountain, Jiangjin, Chongqing (28°39', 106°24')	KY349549(SigmSche17), KY349550(A209_18(f)), KY349551(A209_19)
	16	Kuankuoshui Nature Reserve, Guiyang, Guizhou (28°14',107°12')	KY349538(A5909_102)
	38	Zijin Mountain, Nanjing, Jiangsu (32°04', 118°51')	KY349560(A209_102)
*Sigmella* sp.1	42	Hejiaping, Changyang, Hubei (30°17', 110°34')	KY349533(SigmSp11), KY349534(SigmSp12), KY349535(SigmSp13)
*Sigmella* sp.2	19	Shengtang Mountain, Jinxiu, Guangxi (23°58', 110°07')	KY349531(SigmSp21), KY349532(62A3_1)
*Sigmella* sp.3	38	Zijin Mountain, Nanjing, Jiangsu (32°04', 118°51')	KY349536(SigmSp31), KY349537(62A2_1)
*Sorineuchora nigra*	39	Fuxi Village, Huangshan, Anhui (30°04', 118°09')	KY349516(SoriNigr1), KY349517(A15_12(f)), KY349517(A15_13(f))
	9	Emei Mountain, Leshan, Sichuan (29°34', 103°26')	KY349519(SoriNigr2)
	41	Qingtaiguan, LuoTian, Hubei (31°11', 115°41')	KY349520(SoriNigr3), KY349521(A15_32), KY349522(A15_33)
*Sorineuchora bivitta*	17	Zhuoshui, Qiangjiang, Chongqing (29°18', 108°46')	KY349592(SoriBivi1)
	25	Diaoluo Mountain, Lingshui, Hainan (18°43', 109°52')	KY349593(SoriBivi2)
*Symploce evidens*	24	Qixianling Forest Park, Baoting, Hainan (18°41', 109°40')	KY349670(SympEvid1), KY349671(SympEvid2), KY349672(SympEvid3)
	23	Wuzhishan Nature Reserve, Wuzhishan, Hainan (18°53', 109°40')	KY349673(SympEvid4(f)), KY349674(A3109_42(f)), KY349675(A3109_43(f))
*Symploce* sp.1	17	Zhuoshui, Qiangjiang, Chongqing (29°18', 108°46')	KY349691(SympSp11)
	18	Gongtan, Youyang, Chongqing (28°56', 108°21')	KY349692(SympSp12)
*Symploce* sp.2	36	Tongmu, Wuyishan, Fujian (27°45', 117°41')	KY349689(SympSp21), KY349690(62A8_1)
*Symploce* sp.3	19	Shengtang Mountain, Jinxiu, Guangxi (23°58', 110°07')	KY349693(SympSp31), KY349694(62A9_1)
*Symploce torchaceus*	25	Diaoluo Mountain, Lingshui, Hainan (18°43', 109°52')	KY349695(SympTorc2), KY349696(SympTorc3), KY349697(A4710_23)
	22	Jianfengling Forest Park, Ledong, Hainan (18°44', 108°50')	KY349698(A3809_32(f))
*Symploce* sp.4	23	Wuzhishan Nature Reserve, Wuzhishan, Hainan (18°53', 109°40')	KY349699(SympSp41), KY349700(62A7_1)
*Symploce wulingensis*	20	Longtan Forest Park, Guiping, Guangxi (23°31', 109°59')	KY349701(SympWuli2), KY349702(A3809_22(f))
	22	Jianfengling Forest Park, Ledong, Hainan (18°44', 108°50')	KY349706(SympWuli3), KY349707(A3809_33(f))
	25	Diaoluo Mountain, Lingshui, Hainan (18°43', 109°52')	KY349705(SympWuli4)
	34	Taimu Mountain, Fuding, Fujian (27°11', 119°57')	KY349703(SympWuli5)
	36	Tongmu, Wuyishan, Fujian (27°45', 117°41')	KY349704(SympWuli6(f))
*Symplocodes mamubria*	1	Nabang, Yinjiang, Yunnan (24°45', 97°34')	KY349569(SympMamu1), KY349570(SympMamu3(n))
	1	Nabang, Yinjiang, Yunnan (24°45', 97°34')	KY349568(SympMamu4)

Sampling localities of Ectobiidae spp. used in this study. The letter n after the voucher means the sample is nymph, f means the sample is female.

### Morphological types

We checked all the specimens, then the specimens of male adults were morphologically identified into species where possible or species indet (represented by sp.1, sp.2 and so on). Standard morphological characters were chosen to identify the specimens as follows: presence or absence of pulvilli and arolia, spinal type on anterior-ventral margin of fore-femur, the claws distinctly toothed or not, the degree of wing development (i.e., the number of incomplete branches and the area of appendicular field of hind wing), overall body shape, characteristics of male genitalia and variation of abdominal tergal glands [9,46–55].

Within each species or species indet, we did not genetically sample each individual but chose the male individual form different localities for barcoding in order to obtain more genetic diversity. At the same time we also attempted to sample different variants within the same types to make sure that we could uncover new diversity. Specimens of female adults and nymphs were not delimited but used directly for PCR analysis and DNA sequencing.

### DNA extraction, PCR and sequencing

The hind legs were used for molecular studies, and the other body parts were stored in 95% ethanol as voucher specimens. In total, 297 cockroach specimens were used for COI sequencing in this study (Table 1). All specimens were deposited into the College of Plant Protection, Southwest University, Chongqing, China.

Total DNA was preserved in 100% ethanol and stored at −20°C. The extraction procedure was according to the TIANamp Genomic DNA Kit (Tiangen Biotech, Beijing). Fragments of COI were amplified using PCR. Primers for the amplifications are LCO1490 (5'—GGT CAA CAA ATC ATA AAG ATA TTG G—3') and HCO2198 (5'—TAA ACT TCA GGG TGA CCA AAA AAT CA—3') [56]. The amplification conditions were: initial denaturation at 94°C for 3 or 5 min, followed by 35 cycles of 30 s at 94°C, 30 s at 45°C- 49°C, and 1 min at 72°C, and final extension of 10 min at 72°C. The 35 μl PCR reaction mixture included 19.95 μl of ultrapure water, 3.5 μl of 10× PCR buffer (Mg^2+^ Free), 2.8 μl of dNTP mixture (2.5 mM), 2.8 μl of MgCl_2_ (25mM), 1.4 μl of each primer (10 μM), 0.35 μl of Taq polymerase (5 U), and 3 μl of the DNA template. The PCR amplification products were tested by electrophoresis on 1% agarose gel containing Godview-II. The successful PCR products were sent for sequencing at the BGI Technology Solutions Company Limited (BGI-Tech) (Beijing, China) using the aforementioned primers. All sequences were deposited at the National Center for Biotechnology Information (NCBI) GenBank (Table 1).

### Sequence alignment and phylogenetic analysis

A total of 314 COI sequences were analyzed. This included 297 sequences from this study (Table 1), 5 sequences representing 4 species of Ectobiidae cockroaches downloaded from GenBank; 10 sequences representing 10 species of Blaberidae cockroaches downloaded from GenBank; and 2 mantid sequences (outgroups: *Bantia werneri* and *Hoplocorypha* sp) (Table 2). Sequences were aligned using MUSCLE 3.8 [58]. Among our 297 sequences, 115 identical COI haplotypes were found and removed from the analysis. Intraspecific and interspecific genetic divergence values are quantified based on the Kimura 2-parameter (K2P) distance model [59], using MEGA 6.06 [60].

**Table 2:** 

Species	Family	Reference	Accession Number
*Epilampra* sp.	Blaberidae	[61]	EU253831
*Zetobora* sp.	Blaberidae	[62]	KF372540.1
*Pycnoscelus* sp.	Blaberidae	[63]	KF155021
*Rhabdoblatta marginata*	Blaberidae	[39]	KF640068
*Geossapheus dilatatus*	Blaberidae	[64]	HQ936976
*Rhabdoblatta bielawskii*	Blaberidae	[39]	KF640067
*Minablatta* sp.	Blaberidae	[65]	KP986424
*Gromphadorhina portentosa*	Blaberidae	[66]	KM577153
*Parasphaeria boleiriana*	Blaberidae	[61]	EU253832
*Macropanesthia kinkuna*	Blaberidae	[64]	HQ936979
*Supella longipalpa*	Ectobiidae	[66]	KM577124
*Blattella germanica*	Ectobiidae	[67]	JQ350728
*Blattella germanica*	Ectobiidae	[68]	NC_012901
*Phyllodromica iberica*	Ectobiidae	[37]	AM600685, AM600690
*Bantia werneri*	Thespidae	[69]	FJ802796.1
*Hoplocorypha* sp.	Thespidae	[69]	FJ802828

Blaberoidea and Mantids (Outgroups) Used in This Study and GenBank Accession Number.

To explore phylogenetic relationships among these closely related species, Maximum likelihood (ML) and Bayesian inference (BI) analyses were performed using RAxML 7.7.1 [70] and MrBayes 3.2 [71] respectively. For ML, the GTRGAMMA model was selected for the COI datasets and 1000 bootstrap replicates were performed. For BI, we selected the nucleotide substitution model of COI according to the Bayesian Information Criterion (BIC) in ModelGenerator v.0.851 [72]. The best-fit model for COI was GTR+I+G. Two independent sets of Markov chains were run, each with one cold and three heated chains for 1×10^7^generations, and every 1000^th^ generation was sampled. Convergence was inferred when a standard deviation of split frequencies <0.01 was completed. Sump and sumt burninfrac was set to 25% and contype was set to allcompat.

### Divergence date analyses

We also performed divergence date analyses to infer the evolution of Ectobiidae. For this analysis, the best-fitting models were chosen as follows: Codon position 1: SYM+G, Codon position 2: K81uf+I+G and Codon position 3: TrN+G using PartitionFinder V1.1.1. The molecular clock was calibrated using six minimum age constraints based on cockroach fossils as shown in Table 3. Analyses were performed using a relaxed molecular-clock model using the Bayesian phylogenetic software BEAST 1.8.1 [73]. Rate variation was modeled among branches using uncorrelated lognormal relaxed clocks [73], with a single model for all genes. A Yule speciation process was used for the tree prior [74] and posterior distributions of parameters, including the tree, were estimated using MCMC sampling. We performed two replicate MCMC runs, with the tree and parameter values sampled every 5,000 steps over a total of 50 million generations. A maximum clade credibility tree was obtained using TreeAnnotator within the BEAST software package with a burn-in of 1,000 trees. Acceptable sample sizes and convergence to the stationary distribution were checked using Tracer 1.5 [73].

**Table 3:** 

Species	Age (Ma)/ Minimum Age Constraint for Group	Calibration Group	Soft Maximum Bound (97.5% probability)	Reference
*Juramantis initialis*	145	cockroaches +mantids	250	[75]
*Piniblattella sharingolensis*	125	cockroaches	250	[76]
*Epilampra* sp.	41.3	*Epilampra* +*Rhabdoblatta* + *Parasphaeria*	130	[77]
*Pycnoscelus gardneri*	41.3	*Pycnoscelus* +*Minablatta*	130	[78]
*Zetobora brunneri*	33.9	*Zetobora* + its sister	130	[79]
*Supella miocenica*	15.97	*Supella* + its sister	150	[80]

Fossils Used for Estimation of Divergence Time of Major Clades in the Analysis of Ectobiidae with 2 mantid outgroups.

### GMYC analyses

Species, defined as independently evolving lineages, were delimited using the GMYC approach [29]. Time-resolved gene trees were estimated in BEAST 1.8.1 [73] under a strict clock model with the mean clock rate fixed to 1, and using the randoming starting tree. The Birth-Death speciation was used as a tree prior on divergence times. This is an appropriate choice of tree prior because the GMYC approach uses the constant-size coalescent as a null model for hypothesis testing (see[81]). We then applied the single-threshold GMYC method to the ultrametric gene tree generated by BEAST using the SPLITS package [82] in R [83]. The groups delimited were compared to a one-species null model using a likelihood ratio test.

### Evaluating the two methods to delimt species

We used the GMYC result combined with morphological evidence in understanding species limits among ectobiid cockroaches. If GMYC species confromed to the morphospecies that we identified based on morphological data, we could conclude that our original grouping is one species. As to the females and nymphs, we also considered that if they grouped monophyletically with the correspongding males in the BI and ML inferences. But if GMYC result was inconsistent with the morphological result, we checked the specimen again especially the morphological divergence in genitalia to verify species delimitation.

## Results

### COI sequence variation

In this study, the sequenced length of COI excluding the primer was approximately 658bp. All 297 sequences have been deposited in GenBank with accession numbers KY349516 to KY349812 for COI. The COI sequences that we sequenced have high AT content (65.4%), with an average nucleotide composition of A = 29.6%, T = 35.8%, C = 18.4%, and G = 16.2%. Sequence analysis revealed that 313 sites were variable, of which 289 were parsimony informative.

### Phylogenetic inference

**Fig 2:**
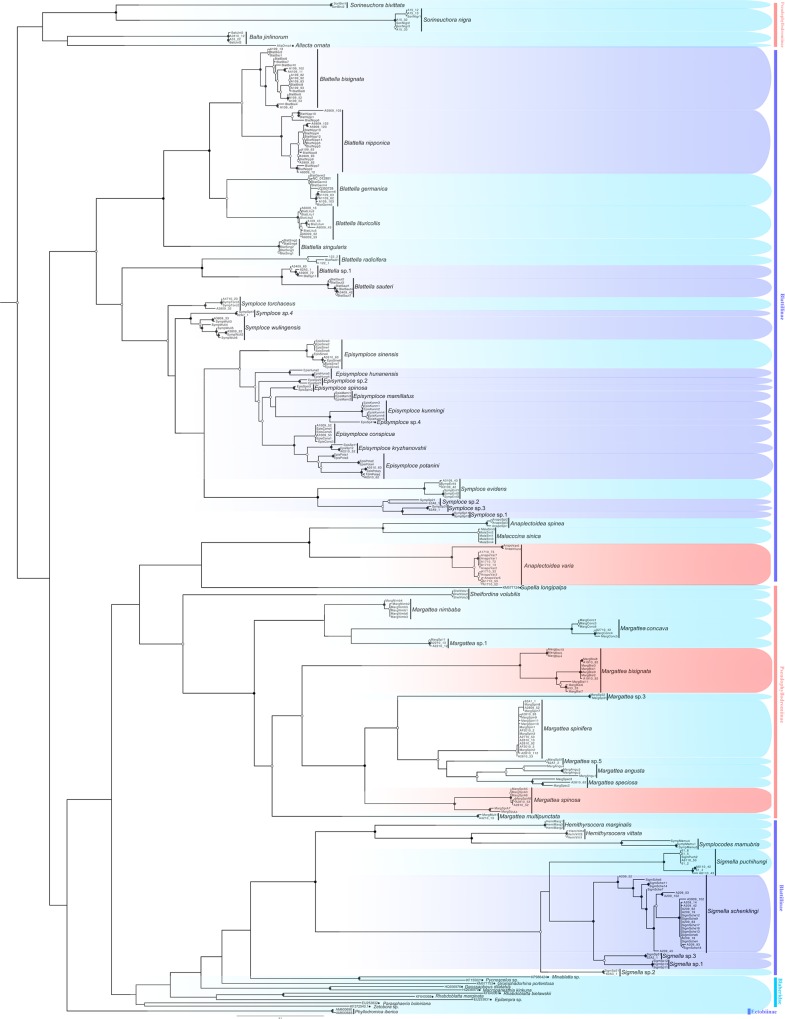
Maximum likelihood (ML) tree derived from COI gene. ● near nodes indicating both BPP and MLB > 90, ■ representing only BPP >90 and ○ representing only MLB >90. Outgroups were not shown.

For COI, phylogenetic constructions yielded similar topologies for the two methods utilized (Fig 2 and S1 Fig). Females and nymphs formed monophyletic groups with their males as recovered in BI and ML analyses (Fig 2, S1 Fig). Most members of one genus were clustered together, with a few exceptions (*Anaplectoidea* and *Symploce*). Members of *Sorineuchora*, *Episymploce*, *Margattea* and *Sigmella* each formed monophyletic groups with high support values. Ectobiidae was found to be paraphyletic with respect to Blaberidae, although support for this grouping was low, and indeed support among the deeper branches of the tree was generally poor. The ectobiid *Phyllodromica iberica* was recovered to be the sister of Blaberidae + partial Blattillinae, although support for this grouping was not strong. Two subfamilies of Ectobiidae, Blattillinae and Pseudophyllodromiinae were found to be paraphyletic, although they were not well supported.

### Divergence date analyses

**Fig 3:**
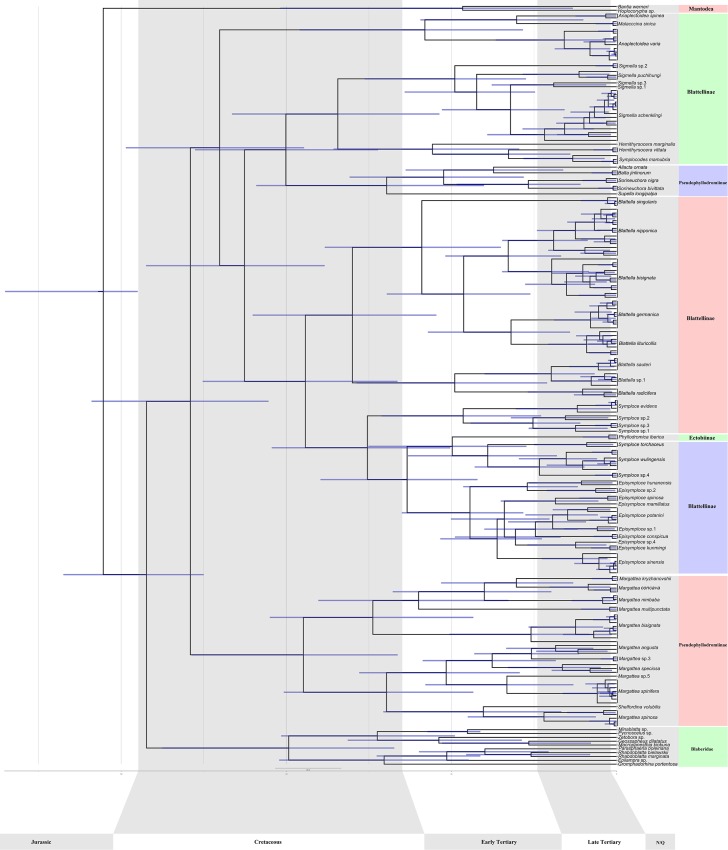
Phylogenetic chronogram of ectobiid species based on COI gene with 2 mantid outgroups, reconstructed using BEAST. An optimal partitioning scheme was determined by PartitionFinder. Scale bar estimates age in millions of years and blue bars represent 95% highest posterior density intervals for the node age.

The timescale for evolution of ectobiid species diversification based on COI and calibrations based on 6 cockroach fossils is shown in Fig 3. The divergence of the lineages leading to mantids and cockroaches was estimated to have occurred 155.41 Ma (145.0–185.09 Ma 95% CI). Blaberidae and Ectobiidae were both found to be monophyletic in this analysis, and were estimated to have diverged 142.3 Ma (125–167.4 Ma 95% confidence interval [CI]). Pseudophyllodromiinae was found to be polyphyletic, having first diverged from other ectobiids ~129 Ma (105.39–159 Ma 95% CI). Blattellinae, which was polyphyletic with respect to Ectobiinae and Pseudophyllodromiinae, first emerged about 120 Ma (94.6–148.5 Ma 95% CI). *Blattella* was found to be monophyletic in this analysis and began to diverge 79.9 Ma (54.59–110.15 Ma). *Blattella germanica* split up from *Blattella lituricollis* 31.96 Ma (14.12–57.10 Ma). The lineages leading to most ectobiid species diverged from their sister lineages around 10 Ma or more.

### GMYC analysis

The likelihoods of the null and GMYC models were 1077.505 and 1129.322 respectively. The GMYC was an improvement over the null model, and was clustered into 53 (confidence interval: 37–65) entities (likelihood ratio = 103.63) including 14 downloaded species.

### Morphological delimitation of chinese ectobiid cockroaches

On the basis of morphological characters, we were able to identify 55 morphospecies of cockroaches among the 297 samples that we examined. These included species from 13 genera of two subfamilies Pseudophyllodromiinae (*Allacta*, *Balta*, *Sorineuchora*, *Shelfordina* and *Margattea*), and Blattellinae (*Malaccina*, *Anaplectoidea*, *Blattella*, *Symploce*, *Symplocodes*, *Episymploce*, *Hemithyrsocera* and *Sigmella*).

### Evaluating the two methods to delimt species

**Fig 4:**
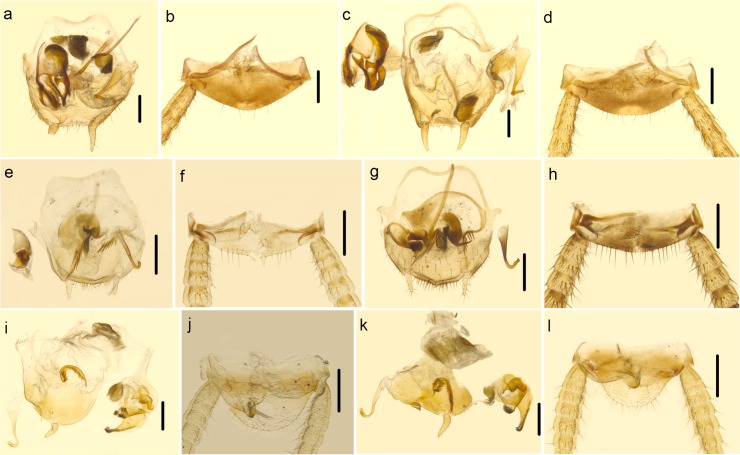
a–b *Margattea bisignata*. a. subgenital plate and genitalia, dorsal view (the left styli and right cercus lost). b. supra-anal plate, ventral view. c–d. *Margattea bisignata*. c. subgenital plate and genitalia, dorsal view. d. supra-anal plate, ventral view. e–f. *Margattea spinosa*. e. subgenital plate and genitalia, dorsal view. f. supra-anal plate, ventral view. g–h *Margattea spinosa*. g. subgenital plate and genitalia, dorsal view. h. supra-anal plate, ventral view. i–j *Anaplectoidea varia*. i. subgenital plate and genitalia, dorsal view. j. supra-anal plate, ventral view. k–l *Anaplectoidea varia*. k. subgenital plate and genitalia, dorsal view. l. supra-anal plate, ventral view. Scale bars (a–l) = 0.5cm.

**Fig 5:**
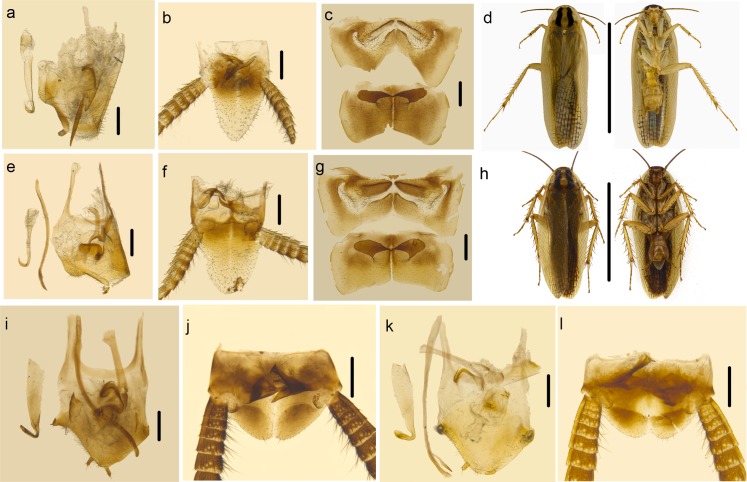
a–d *Blattella nipponica*. a. subgenital plate and genitalia, dorsal view. b. supra-anal plate, ventral view. c. the seventh and eighth abdominal tergum, dorsal view. d. habitus. e–h *Blattella bisignata*. e. subgenital plate and genitalia, dorsal view. f. supra-anal plate, ventral view. g. the seventh and eighth abdominal tergum, dorsal view. h. habitus. i–j *Blattella* sp.1 i. subgenital plate and genitalia, dorsal view. j. supra-anal plate, ventral view. k–l *Blattella sauteri*. k. subgenital plate and genitalia, dorsal view. l. supra-anal plate, ventral view. Scale bars (a–c, e–g, i–l) = 0.5cm, (d, h) = 1.0cm.

**Fig 6:**
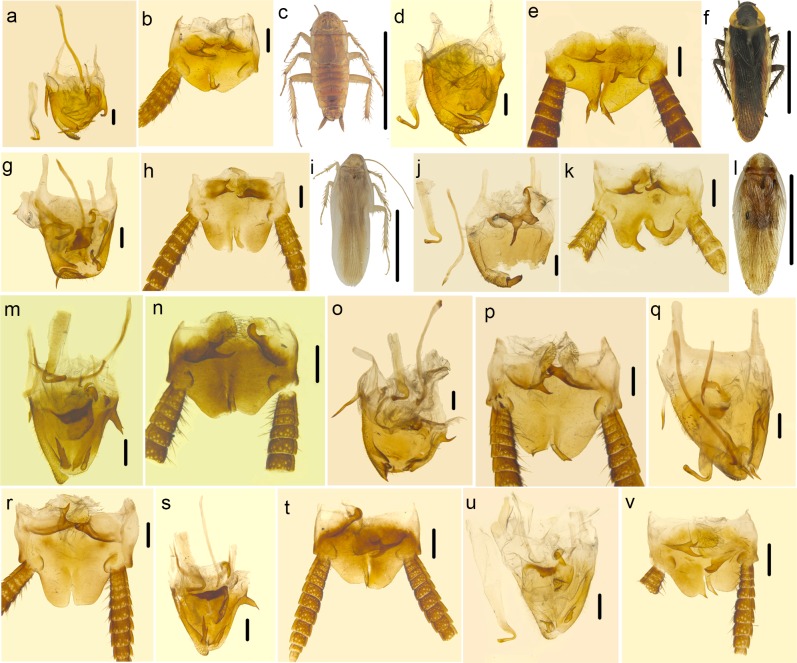
a–c *Episymploce* sp.2. a. subgenital plate and genitalia, dorsal view. b. supra-anal plate, ventral view, c. habitus. e–f *Episymploce hunanensis*. e. subgenital plate and genitalia, dorsal view. d. supra-anal plate, ventral view, f. habitus. g–h *Episymploce mamillatus*. g. subgenital plate and genitalia, dorsal view. h. supra-anal plate, ventral view, i. habitus. j–l *Episymploce spinose*. j. subgenital plate and genitalia, dorsal view. k. supra-anal plate, ventral view, l.♀, habitus. m–n *Episymploce kryzhanovshii*. m. subgenital plate and genitalia, dorsal view. n. supra-anal plate, ventral view. o–p *Episymploce conspicua*. 0. subgenital plate and genitalia, dorsal view. p. supra-anal plate, ventral view. q–r *Episymploce potanini*. q. subgenital plate and genitalia, dorsal view. r. supra-anal plate, ventral view. s–t *Episymploce* sp.4. s. subgenital plate and genitalia, dorsal view. t. supra-anal plate, ventral view. u–v *Episymploce kunmingi*. u. subgenital plate and genitalia, dorsal view. v. supra-anal plate, ventral view. Scale bars (a–b, e–d, g–h, j–k, m–v) = 0.5cm, (c, f, I, l) = 1.0cm.

**Fig 7:**
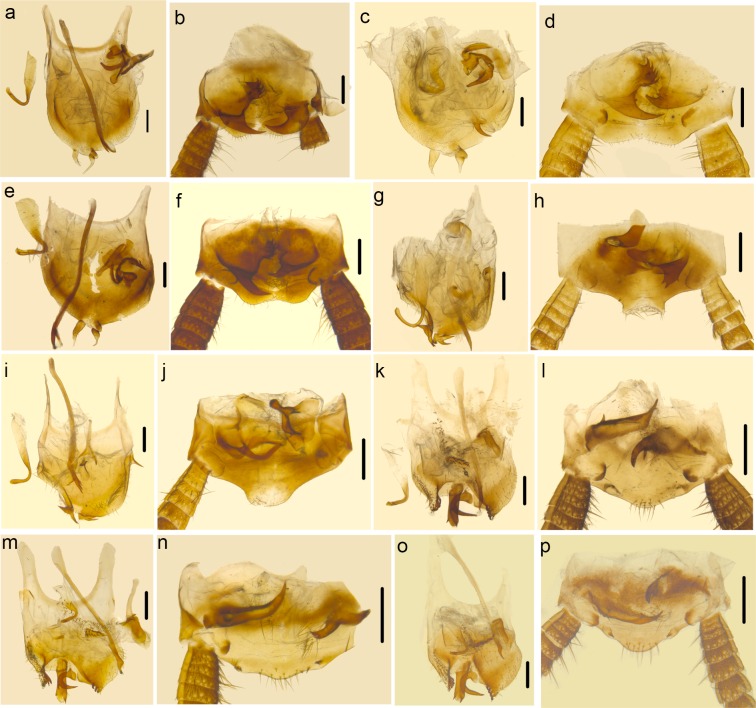
a–b *Symploce* sp.2. a. subgenital plate and genitalia, dorsal view. b. supra-anal plate, ventral view. c–d *Symploce* sp.1. c. subgenital plate and genitalia, dorsal view. d. supra-anal plate, ventral view. e–f *Symploce* sp.3. e. subgenital plate and genitalia, dorsal view. f. supra-anal plate, ventral view. g–h *Symploce* sp.4. g. subgenital plate and genitalia, dorsal view. h. supra-anal plate, ventral view. i–j *Symploce wulingensis*. i. subgenital plate and genitalia, dorsal view. j. supra-anal plate, ventral view. k–l *Sigmella* sp.3. k. subgenital plate and genitalia, dorsal view. l. supra-anal plate, ventral view. m–n *Sigmella schenklingi*. m. subgenital plate and genitalia, dorsal view. n. supra-anal plate, ventral view (the left cercus lost). o–p *Sigmella* sp.1. k. subgenital plate and genitalia, dorsal view. l. supra-anal plate, ventral view. Scale bars (a–n) = 0.5cm.

There is a difference in species delimitation between the two methods. The COI GMYC groups of our samples partly corresponded to the 28 ectobiid species with light blue highlights in Fig 2. Based on genitalia information (Fig 4) and genetic distance, *Anaplectoidea varia*, *Margattea bisignata* and *Margattea spinosa* with light red highlights in Fig 2 were split into 2 different morphospecies respectively; but the GMYC result suggested that they are representatives of 3 different species. According to GMYC result, *Blattella nipponica* and *Blattella bisignata*, *Symploce* sp.1, *Symploce* sp.2 and *Symploce* sp.3, *Episymploce hunanensis*, *Episymploce spinosa*, *Episymploce* sp.2 and *Episymploce mamillatus*, *Episymploce kryzhanovshii*, *Episymploce conspicua* and *Episymploce potanini*, *Episymploce* sp.4 and *Episymploce kunmingi*, *Symploce* sp.4 and *Symploce wulingensis*, *Blattella* sp.1 and *Blattella sauteri*, *Sigmella* sp.1, *Sigmella* sp.3 and *Sigmella schenkling*, with light purple highlights in Fig 2, were separately categorized into the same morphotype; but based on morphological information, they were all treated different species (Figs 5–7). As to the incongruence, we checked the specimens again to make sure that the morphological divergence in genitalia was the intraspecific difference or not. Finally, the variations between different morphospecies of *Anaplectoidea varia*, *Margattea bisignata* and *Margattea spinosa* (light red highlights in Fig 2) was determined as intraspecific difference although large genetic distance existed, whereas the variations among other morphospecies (light purple highlights in Fig 2) was determined as interspecific difference although maybe there was slight genetic distance between them.

Based on the combination of GMYC result with morphological information, 52 ectobiid species were delimited successfully. The intraspecific and interspecific sequence divergence ranged from 0.0 to 7.0% and 4.6 to 30.8%, respectively.

## Discussion

The number of species recovered from our GMYC analyses (53 entities (confidence interval: 37–65)) partly conformed to the number of morphological species that we identified (69 species, including 55 ectobiid species). Finally 52 ectobiid species were delimited successfully using GMYC method with morphological information. GMYC method exhibited a significant reduction (13 species) in total species count. Our results therefore show that DNA based species delimitation methods perform not well for cockroaches, just as a complementary method to species delimitation based on morphological data. Certainly, DNA based identification methods are especially useful for cockroaches, due to a lack of defining characters among females and nymphs of these organisms. Our study is the first attempt to investigate species delimitation of a large number of cockroach species, which included females and nymphs. GMYC employing a tree based on COI helped us to accurately identify all 52 ectobiid morphospecies. Thus, phylogenetic analysis of COI in combination with GMYC proved to be an invaluable tool for delimiting cockroach species and complementing classical taxonomy in the context of effective identification.

### Species delimitation

For the ectobiid cockroaches studied here, the intraspecific and interspecific K2P genetic divergence ranged from 0.0 to 7.0% and 4.6 to 30.8%, which more or less similar to other groups (*e*.*g*. thrips: 0.0 to 7.91% and 8.65% to 31.15% [84]; mosquitoes: 0–1.67% and 2.3–21.8% [85]), although the greater intraspecific diversity showed some overlap with interspecific divergence. Hebert *et al*. [86] proposed that the genetic divergence cutoff for species identification should be at least 10 times greater than within species. However, many exceptional cases that do not follow this proposal have been reported. Barcode sequence divergence in conspecific specimens ranged from 0–1.67% and congeneric species showed from 2.3–21.8% divergence for 122 mosquito species in China [85], while for 21 mosquito species in Pakistan, these values were from 0–2.4% and 2.3–17.8% divergence [87]. Rebijith *et al*. [84] reported that the intraspecific and intrageneric distances of COI barcode sequence for 151 thrip species ranged from 0.0 to 7.91% and 8.65% to 31.15% respectively. Both Meyer & Paulay [88] and Wieners *et al*. [89] proposed that the “barcoding gap” was an artifact of insufficient sampling across taxa. In other words, if sufficient sampling were undertaken, intraspecific variation would overlap with interspecific divergence.

Although the COI GMYC groups of our samples partly corresponded to the 28 ectobiid species, the male adults of one species have the same morphological and genitalia characters, but for females or nymphs, it may be not the same as males and only DNA based methods can be used to solve it in that case in general. Females and nymphs formed monophyletic groups with their males as recovered in BI and ML analyses (Fig 2, S1 Fig), consistent with the results from GMYC method.

Although genital morphology has been proved to be more effective in diagnosing cockroaches, it is also very challenging to use it in the taxonomy. *Anaplectoidea varia* (Fig 4I–4L), *Margattea bisignata* (Fig 4A–4D) and *Margattea spinosa* (Fig 4E–4H) were each split into 2 different morphospecies because of their difference in genitalia. After careful examination, the variations between different morphospecies of three species listed above was determined as intraspecific difference. *Anaplectoidea varia* (the genetic distance of AnapoVar4, 6 (Fig 4I and 4J) vs. others (Fig 4K and 4L): 6.3%), *Margattea bisignata* (the genetic distance of MargBisi4 (Fig 4A and 4B) vs. others (Fig 4C and 4D): 6.0%) and *Margattea spinosa* (the genetic distance of MargSpiA4, 7 (Fig 4E and 4F) vs. others (Fig 4G and 4H): 7.8%) with light red highlights in Fig 2 all showed slight difference in genitalia and large genetic distance, much higher than that of other intraspecific divergences, even higher than interspecific genetic divergences (4.6%) between some species (*Episymploce kunmingi* and *Episymploce* sp.4, *Episymploce kryzhanovshii* and *Episymploce conspicua*). The sample localities for *Margattea bisignata* were geographically distant from each other (~1000 km); MargBisi4 was from E’mei, Sichuan Province while other members were from Guangxi Province, Guangdong Province, Fujian Province and Hubei Province. The samples of AnapoVar4, 6 were from Hainan Province, which is an isolated island separated by Qiongzhou Strait from mainland. So gene flow might be hindered between the two geographically distant populations, which accounts for the larger intraspecific divergences of *Margattea bisignata* and *Anaplectoidea varia*. But for *Margattea spinosa*, all samples were from Guangxi and Hainan Province, which are the tropical regions in China. That the tropical and subtropical taxa had the greater diversity and substantial phylogeographic structure [90] maybe resulted to increase intraspecific genetic divergence.

The similar pairs *Episymploce kunmingi* and *Episymploce* sp.4 was delimited as one GMYC species but each recovered as single group in BI and ML inference (Fig 2, S1 Fig). On the other hand, the genetic distance between *Episymploce kunmingi* (Fig 6: u-v) and *Episymploce* sp.4 (Fig 6S and 6T) was only 4.6%, yet there were strong morphological differences between them as follows: (1) the former with minute spines present in the right margin of subgenital plate, but in the latter, large spines present; (2) the right style shorter than the left one in *Episymploce kunmingi*, but for *Episymploce* sp.4, the right style distinctly longer than the left one; (3) *Episymploce kunmingi* with a spinelike process near the right side of excavation, but *Episymploce* sp.3 without any process near the excavation. After we checked the morphological characters including the male genitalia, we were unable to find differences between them. The genetic distance between *Episymploce conspicua* and *Episymploce kryzhanovshii* was also 4.7%, yet *Episymploce conspicua* (Fig 6O and 6P) was clearly distinguished from *Episymploce kryzhanovshii* (Fig 6M and 6N) by the following characters: (1) body of *Episymploce conspicua* medium, about 2.2 cm including tegmina, but in *Episymploce kryzhanovshii*, body small and about 1.1 cm including tegmina; (2) posterior margin of supra-anal plate with a V-shaped concavity at middle and symmetrical in *Episymploce conspicua*, only a shallow crack present in *Episymploce kryzhanovshii* and asymmetrical; (3) lateral margin of genital plate with apex tapering and without spines scattered in *Episymploce conspicua*, but in *Episymploce kryzhanovshii*, apex rounded and scattered with spines; (4) spines absent in both styli of *Episymploce conspicua*, but present in *Episymploce kryzhanovshii*.

Although the genetic distance between them was 6.7%, *Blattella nipponica* (Fig 5A–5D) and *Blattella bisignata* (Fig 5E–5H) show considerate divergence in color and body shape, even more conspicuous in male genitalia and 7^th^-8^th^ tergites. Similarly, the genetic distance among four closely related species, *Episymploce hunanensis* (Fig 6D–6F), *Episymploce spinosa* (Fig 6J–6L), *Episymploce* sp.2 (Fig 6A–6C) and *Episymploce mamillatus* (Fig 6G and 6H), which were delimited into one GMYC species, is from 6.9% to 9.2%. Especially *Episymploce* sp.2 (Fig 6C) is typically brachypterous and distinguished from the left three species. These results indicate that morphological differentiation can occur despite low genetic differentiation. Only using morphological data combined with GMYC method, ectobiid species could be delimited successfully.

### Phylogeny and evolutionary timescale of ectobiidae

Blaberoidea includes the groups Ectobiidae (Pseudophyllodromiinae, Blattellinae, Ectobiinae, Nyctoborinae) and Blaberidae [65,91]. These groups have been shown to form a monophyletic group by morphological [92,93] and molecular [65,94–98] data; in most previous studies, Ectobiidae was recovered as paraphyletic with respect to Blaberidae. In our study, we obtained the clade Blaberoidea with high support values (BPP = 100, MLB = 100) based on substantial cockroach COI samples on a large scale; however, we had no samples from Nyctoborinae. Although our analyses recovered each as paraphyletic (see below), it should be noted we only used one mitochondrial gene, which is likely to be less reliable compared with the multi-gene analyses of other studies.

Grandcolas [92] proposed that Pseudophyllodromiinae was monophyletic and the sister group of Blaberidae. Klass [99,100] and Klass & Meier [93] considered the Pseudophyllodromiinae to be paraphyletic, while Inward *et al*. [94] obtained a monophyletic Pseudophyllodromiinae as sister group of Ectobiinae. In our study, Pseudophyllodromiinae was paraphyletic and one part of Pseudophyllodromiinae (*Allacta*, *Balta*, *Sorineuchora*) was recovered to be the sister group of the left Blaberoidea members (MLB>90).

The trees based on BI and ML analyses show that the members of genus *Anaplectoidea* was not clustered together; on the contrary, *Anaplectoidea spinea* and *Malaccina sinica* formed a monophyletic group (BPP = 99, MLB = 100), which was the sister to other members of *Anaplectoidea* (BPP = 100, MLB = 100). These two genera are highly morphologically similar, and the differences between these two genera mainly manifest in the numbers of incomplete branches and the area of appendicular field of hind wings according to the morphology. Roth [53] transferred two species of *Anaplectoidea* to *Malaccina*. *Anaplectoidea* and *Malaccina* should probably be treated as one genus because of their close genetic relationship.

The relationships between *Hemithyrsocera* and *Symplocodes*, *Episymploce* and *Symploce* were similar to those of *Anaplectoidea* and *Malaccina*. The only character that clearly separates *Symplocodes* from *Hemithyrsocera* is the distinctly toothed tarsal claws in the former [52]. *Episymploce* and *Symploce* are highly morphologically similar, and the main differences between them are the symmetry of the supra-anal plate and the thickness of lateral margins in subgenital plate. Wang & Che [1] suggested that *Symploce wulingensis* should be transferred to *Episymploce*. It is possible that the genus delimitation is only an artifact of cockroach taxonomy and that they are not a natural group. In that case, the genera mentioned above would need critical revision.

*B*. *germanica* and a number of other *Blattella* spp. (*B*. *singularis*, *B*. *lituricollis*, *B*. *bisignata and B*. *nipponica*) clustered together with high supported (BPP = 100, MLB = 100). They all belong to the *germanica* species-group and resemble each other in morphology. They are so similar externally that a large number of other species have been wrongly regarded as the German cockroach in China. However *Blattella germanica* appears to be restricted to buildings, vehicles and ships as an important pest in China, while other members are found in leaf litter and grass or shrubs in forested area [101]. *Blattella radicifera*, *Blattella* sp.1 and *Blattella sauteri* formed a separate clade from other *Blattella* in our analysis. They are clearly distinguished from other *Blattella* members by short and broad supra-anal plate (Fig 5J and 5L) (while in other *Blattella* members, supra-anal plate is tongue-shaped (Fig 5B and 5F)).

Blaberidae was not recovered to be a monophyletic group but formed a monophyly with partial Blattellinae (*Symplocodes*, *Hemithyrsocera* and *Sigmella*). This was not consistent with other recent studies [65,91,102], which revealed that Blaberidae was monophyletic.

The present study is the first to provide fossil calibrated molecular estimates of divergence time for the major lineages of Ectobiidae based on a wide variety of taxa, although the dates should be interpreted with caution due to the use of only a single mitochondrial marker. The divergence of Blaberidae and Ectobiidae was estimated to have occurred 142.3 Ma (125–167.4 Ma), largely consistent with previous estimate (Lo *et al*. [103]: ~140–145 Ma; Djernæs *et al*. [91]: ~185 Ma). The major subfamilies of Ectobiidae were found to have diverged between ~125–110 Ma, and most morphospecies pairs were found to have diverged ~10 or more Ma.

## Conclusion

Our results show that GMYC methodology generates species hypotheses for cockroaches that are partly consistent with those based on traditional morphological techniques. However, it’s tenuous to only take GMYC for granted as effectiveness of cockroach species delimitation, despite it performs well for other groups. The GMYC technique shows promise as a rapid, precise, independent identification approach for the discrimination of cockroach species of different life stages and color morphs to some extent. Moreover, as our study has revealed the combination of GMYC method with morphological data to delimit species successfully, the approaches we used may help to increase our understanding of cockroach biodiversity.

## Supporting Information

S1 Fig Bayesian (BI) tree derived from COI gene. Numbers near nodes indicate the percentage of posterior probabilities. Outgroups are not shown.
(TIF)

S1 File License information of the map used in the paper. (DOCX)
