# Supplementary figures and images for: Species Delimitation and Phylogenetic Relationships in Ectobiid Cockroaches (Dictyoptera, Blattodea) from China
Source: PLoS One. 2017 Jan 3;12(1):e0169006. doi: 10.1371/journal.pone.0169006 (PMC5207705; doi:10.1371/journal.pone.0169006)

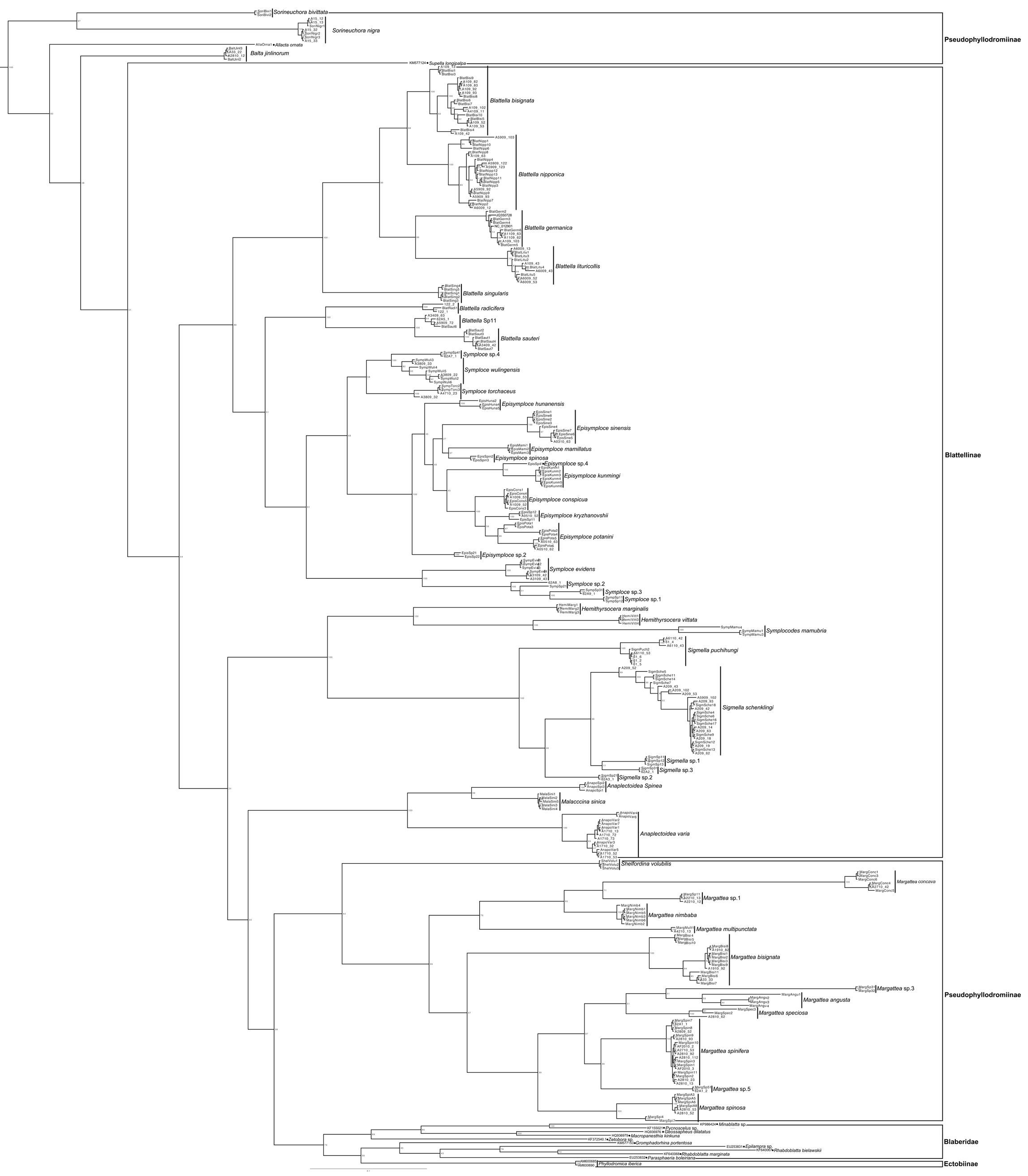

Supplement: S1 Fig — Numbers near nodes indicate the percentage of posterior probabilities. Outgroups are not shown. (TIF) [file pone.0169006.s001.tif]
