# Supplementary material for: Species Delimitation and Phylogenetic Relationships in Ectobiid Cockroaches (Dictyoptera, Blattodea) from China
Source: PLoS One. 2017 Jan 3;12(1):e0169006. doi: 10.1371/journal.pone.0169006 (PMC5207705; doi:10.1371/journal.pone.0169006)
Supplement: S1 File — (DOCX) [file pone.0169006.s002.docx]

The map we used in the manuscript (fig. 1) originates from the author, Chao Li.

This file is licensed under the Creative Commons Attribution-Share Alike 4.0 International license.

<https://commons.wikimedia.org/wiki/File:Blank_maps_of_China.svg>
